# Consequences of Mixotrophy on Cell Energetic Metabolism in *Microchloropsis gaditana* Revealed by Genetic Engineering and Metabolic Approaches

**DOI:** 10.3389/fpls.2021.628684

**Published:** 2021-05-25

**Authors:** Davide Dal Bo, Leonardo Magneschi, Mariette Bedhomme, Elodie Billey, Etienne Deragon, Mattia Storti, Mathilde Menneteau, Christelle Richard, Camille Rak, Morgane Lapeyre, Mehdi Lembrouk, Melissa Conte, Valérie Gros, Guillaume Tourcier, Cécile Giustini, Denis Falconet, Gilles Curien, Guillaume Allorent, Dimitris Petroutsos, Frédéric Laeuffer, Laurent Fourage, Juliette Jouhet, Eric Maréchal, Giovanni Finazzi, Séverine Collin

**Affiliations:** ^1^Université Grenoble Alpes (UGA), Centre National Recherche Scientifique (CNRS), Commissariat Energie Atomique, Energies Alternatives (CEA), Institut National Recherche Agriculture, Alimentation, Environnement (INRAE), Interdisciplinary Research Institute of Grenoble, IRIG-Laboratoire de Physiologie Cellulaire et Végétale, Grenoble, France; ^2^Total Refining Chemicals, Tour Coupole, Paris La Défense, France

**Keywords:** *Microchloropsis gaditana*, mixotrophy, photosynthesis, mitochondrial alternative oxidase, TALE nuclease, lipid metabolism

## Abstract

Algae belonging to the *Microchloropsis* genus are promising organisms for biotech purposes, being able to accumulate large amounts of lipid reserves. These organisms adapt to different trophic conditions, thriving in strict photoautotrophic conditions, as well as in the concomitant presence of light plus reduced external carbon as energy sources (mixotrophy). In this work, we investigated the mixotrophic responses of *Microchloropsis gaditana* (formerly *Nannochloropsis gaditana*). Using the Biolog growth test, in which cells are loaded into multiwell plates coated with different organic compounds, we could not find a suitable substrate for *Microchloropsis* mixotrophy. By contrast, addition of the Lysogeny broth (LB) to the inorganic growth medium had a benefit on growth, enhancing respiratory activity at the expense of photosynthetic performances. To further dissect the role of respiration in *Microchloropsis* mixotrophy, we focused on the mitochondrial alternative oxidase (AOX), a protein involved in energy management in other algae prospering in mixotrophy. Knocking-out the AOX1 gene by transcription activator-like effector nuclease (TALE-N) led to the loss of capacity to implement growth upon addition of LB supporting the hypothesis that the effect of this medium was related to a provision of reduced carbon. We conclude that mixotrophic growth in *Microchloropsis* is dominated by respiratory rather than by photosynthetic energetic metabolism and discuss the possible reasons for this behavior in relationship with fatty acid breakdown via β-oxidation in this oleaginous alga.

## Introduction

Photosynthesis converts light into chemical potential for downstream cell metabolism. Absorbed photons provide the energy required to start electron flow across the photosynthetic (thylakoids) membrane, reducing NADP^+^ to NADPH. Electron flow is coupled with proton accumulation in the thylakoid lumen generating a proton gradient, consumed by the ATP synthase CFo-F1 to synthesize ATP. Both ATP and NADPH are used for CO_2_ assimilation in the Calvin–Benson–Bassham cycle. In C3 plants and algae, three ATP and two NADPH molecules are consumed per CO_2_ assimilated (Eberhard et al., 2008), but the ATP/NADPH ratio generated by linear electron flow (LEF) *in vivo* is probably lower than 1.5 (Allen, 2002). Consequently, it is believed that part of the electrons generated by LEF at the Photosystem I (PSI) acceptor side might be redirected toward alternative sinks other than NADP^+^ to support ATP synthesis without concomitant NADPH generation. This rerouting, which represents a small proportion of the total electron flow, is likely essential to optimize CO_2_ assimilation (reviewed, e.g., in Kramer and Evans, 2011; Johnson and Alric, 2013; Curien et al., 2016; Peltier et al., 2016).

Several processes may divert electrons from LEF. They are mainly classified as cyclic electron flow (CEF) around PSI (Shikanai, 2007) and water-to-tater cycles (WWCs, Asada, 1999, 2000). CEF is a process in which electrons generated at the PSI acceptor side are used to reduce a PSI electron donor (likely the PQ pool), likely via two routes: the PGR5/PGRL1 (Yamori and Shikanai, 2016) and the NADH dehydrogenase-like (NDH) complex-mediated ones (Peltier et al., 2016). WWCs include the photoreduction of O_2_ by PSI (the Mehler reaction) (Mehler, 1951; reviewed in Asada, 1999, 2000) and photorespiration, i.e., the oxygenase activity of Rubisco (reviewed in Ort and Baker, 2002). Metabolic interactions between the chloroplast and mitochondria can also be considered as a WWC (Curien et al., 2016).

In plants, the interaction between chloroplast and mitochondria, mediated by the activity of a malate–oxaloacetate shuttle or of the aspartate–malate shuttle (Noguchi and Yoshida, 2008; Kinoshita et al., 2011) mainly serves the purpose of maintaining redox homeostasis in the cell, with no or small contributions to photosynthesis (Dutilleul et al., 2003; Cardol et al., 2010). On the other hand, energetic exchanges between the two organelles are essential for growth in microalgae, including chlorophytes—*Chlamydomonas reinhardtii* (Lemaire et al., 1988; Cardol et al., 2009; Dang et al., 2014), *Chlorella vulgaris* (Martínez and Orús, 1991)—and diatoms (*Phaeodactylum tricornutum*, *Thalassiosira pseudonana*, *Thalassiosira weissflogii*, *Fragilaria pinnata*, *and Ditylum brightwellii*; Bailleul et al., 2015; Kim et al., 2016). In these organisms, energetic interactions between the organelles are often paralleled by a capacity to grow in mixotrophy, i.e., by simultaneously exploiting sunlight and exogenous organic carbon as energy sources (Johnson and Alric, 2012; Villanova et al., 2017; Cecchin et al., 2018).

In this work, we re-examined the occurrence and possible consequences of mixotrophy in *Microchloropsis gaditana*. This alga, formerly known as *Nannochloropsis*, belongs to the Eustigmatophyceae class within Stramenopiles. It has been extensively exploited for biotechnology purposes due to its ability to accumulate lipid reserves (i.e., possible precursors of biofuel) in replete and, to a larger extent, in nutrient-starved conditions (Converti et al., 2009; Moazami et al., 2012; Liu et al., 2017). Consequences of reduced carbon on *Nannochloropsis* and *Microchloropsis* (including *M. gaditana)* growth have been tested with several organic sources, including glucose (Xu et al., 2004a,b; Fang et al., 2004), glycerol (Sforza et al., 2012), and acetate (Hu and Gao, 2003; Menegol et al., 2019), with sometimes contrasting results. We looked at possible mixotrophic substrates, growing cells in multiwell plates coated with organic compounds (Biolog plates, Bochner, 2003; Stefanowicz, 2006). Among the 192 possible substrates tested, none was able to reproducibly enhance the growth of *Microchloropsis*. On the other hand, addition of the Lysogeny broth (LB) to the inorganic ESAW growth medium increased the growth capacity, boosting respiration while decreasing photosynthetic performances at the same time. To further elucidate the role of respiration in *Microchloropsis* mixotrophy, we targeted the mitochondrial alternative oxidase (AOX1), an enzyme that has a central role in energy management and dissipation of excess redox power in the mitochondria in other Stramenopiles like diatoms (Bailleul et al., 2015; Murik et al., 2019). Knock-out mutants, obtained using the transcription activator-like effector nuclease (TALE-N) technology (Jeon et al., 2017), did not display a particular phenotype in phototrophic conditions. However, they lost the positive effect of LB on growth seen in the WT. The lipid content was less decreased in mutants than in the WT under mixotrophic conditions. Overall, we conclude that mixotrophy has a somehow positive role for *Microchloropsis* growth and that this effect is likely mediated by a respiratory consumption of lipid reserves.

## Materials and Methods

### Cultivation of *Microchloropsis gaditana* Cells, Reagents, Statistics

*M. gaditana* CCMP526 was used in all experiments. All the genotypes employed here were maintained on plates prepared with the f/2 medium, solidified with 1% agar under a 12-h:12-h light/dark regime in the presence (transformed strains) or absence (wild-type strain) of the selective antibiotic zeocin (7 μg⋅ml^–1^). For phenotypic analyses, *M. gaditana* was cultivated in artificial seawater (ESAW) using 10 times enriched nitrogen and phosphate sources (5.49 × 10^–3^ M NaNO_3_ and 2.24 × 10^–4^ NaH_3_PO_4_; Dolch et al., 2017). When grown in batches in 250-ml flasks, a volume of 50 ml of liquid medium was inoculated at 2.5 × 10^6^ cells⋅ml^–1^, and cultivation was achieved under gentle agitation (100 rpm) at 20°C on a 12-h:12-h light (60 μmol photons⋅m^–2^⋅s^–1^)/dark cycle. Cells were counted using a Luna Automated Cell Counter (Logos Biosystem, South Korea). The LB medium employed for mixotrophic growth was prepared with a granulated stock from Roth (Germany, product code 6673.4). All the reagents were of ACS purity grade. Growth curves were fitted with an exponential growth function (using the Origin 9.6 software, Microcal, United States) to evaluate the cell division rates. The same software was used for statistical analysis (ANOVA test).

### Biolog Plate Assessment of Growth and Respiration

We evaluated the impact of several carbon sources on algal growth using Phenotype Biolog MicroArrays Plates (PM01, PM02A, and PM05) to pinpoint possible candidates for mixotrophic cultivation of *M. gaditana*. This assay is based on the use of a 96-well plate containing pre-arrayed substrates such as carbohydrates, amino acids, and carboxylic compounds (Bochner, 2003). One hundred fifty microliters of cells at an initial concentration of 2 × 10^6^ cells ml^–1^ was deposited into every well. Cells were grown at 19°C under 40 μmol of photons m^–2^ s^–1^ with a 12-h dark/12-h light regime. Growth was monitored by reading optical density at 750 nm over a period of 2 weeks using a Tecan plate reader (Tecan, Switzerland). We checked that plates were not contaminated by bacteria by visual inspection under a microscope. Cell number was calculated using a calibration curve. Cell division rates were estimated for every compound as described above. Most promising metabolites were re-evaluated for their impact on growth at a 250-ml flask scale. A phototrophic control was grown in parallel. For liquid cultures (Supplementary Figure 1), acetic, malonic, and fumaric compounds were added in their acidic form at 20 mM. The pH of the medium was maintained at 8.0 during these experiments by buffering it with 40 mM Tris-HCl. The substrates present in the Biolog plates PM01, PM02A, and PM05 employed in this work are listed in Supplementary Dataset 1.

### Design of Vectors Containing MgAOX1-Specific TALE-N Subunits

Transcription activator-like effector nucleases (TALE-N) are restriction enzymes made of two subunits, called here Tal1Ng-R and Tal1Ng-L, engineered by fusing a TAL effector DNA-binding domain to a FokI DNA cleavage domain. The DNA binding domain contains a repeated highly conserved amino acid sequence with divergent amino acids, referred to as the repeat variable di-residue (RVD). The Tal1Ng-L and Tal1Ng-R RVDs were purchased from ThermoFisher GeneArt-TALs and synthetized into their commercial vector. The Tal1Ng-L subunit was designed following established guidelines (Sanjana et al., 2012) to bind to the TGCCCAAGCTGGACCCAAA sequence in the MgAOX1 gene (Naga_100568g3), corresponding to RVD (T)-NN-HD-HD-HD-NI-NI-NN-HD-NG-NN-NN-NI-HD-HD-HD-NI-NI-NI, whereas the Tal1Ng-R subunit was designed to bind specifically to TCCATACGCTGTGGTGCGT, corresponding to RVD (T)-HD-HD-NI-NG-NI-HD-NN-HD-NG-NN-NG-NN-NN-NG-NN-HD-NN-NG. In these RVD sequences, “(T)” indicates that the first binding repeat is provided by the vector. Commercial Tal1Ng-L and Tal1Ng-R sequences (comprising RVDs) were subcloned in recipient vectors pCT5Ng or pCT6Ng, generating *Microchloropsis* TALE-N expression vectors pCT57 and pCT58, respectively. pCT5Ng is a pET15b backbone, which contains a first part with the bleomycin/zeomycin-resistance protein from *Streptoalloteichus hindustanus* (ShBle) under the control of the endogenous extension promoter (UEP) and followed by the fcpA (fucoxanthin chlorophyll a/c binding protein) terminator and a second part with a nuclear localization sequence (NLS) DYKDHDGDYKDHDIDYKDDDDKMAPKKKRKVG IHGVPAA (Sanjana et al., 2012), an HA-tag, N-ter sequence of commercial TAL up to AflII site, a C-ter sequence of commercial TAL from XhoI site under the control of UEP and with the fcpA terminator. pCT6Ng is a pET28b backbone, which contains a first part with the ShBle resistance gene under the control of the UEP and with the fcpA terminator and a second part with an NLS (DYKDHDGDYKDHDIDYKDDDDKMAPKKKRKVGIH GVPAA; Sanjana et al., 2012), a His-tag, N-ter sequence of commercial TAL up to AflII site, a C-ter sequence of commercial TAL from XhoI site under the control of UEP and with the fcpA terminator. NLS, HA-tag, and His-tag are codons optimized to *M. gaditana* using the Kazusa webtool (Codon usage tabulated from the international DNA sequence databases: status for the year 2000^1^, Nakamura et al., 2000).

### Nuclear Transformation of *Microchloropsis gaditana*

Plasmids pCT57 and pCT58 were linearized by digestion with ScaI and column purified using a NucleoSpin gel and polymerase chain reaction (PCR) clean-up kit (Macherey-Nagel) following manufacturer’s instructions. Two micrograms (1 μg of TALE-N left subunit + 1 μg of TALE-N right subunit) of linearized plasmids were electroporated into *Microchloropsis gaditana* as previously described (Dolch et al., 2017). Transformed lines were selected on f/2 plates containing 7 μg ml^–1^ of zeocin. Integration of both TALE-N subunits was assessed by colony PCR with primers TAL-Nterm-Rev (GCAGGTCGCTAAAAGAATCG) and TAL-HA-Fw (CCCCGACTACGCTAGCG) for TALEN left subunit and TAL-Nterm-Rev (GCAGGTCGCTAAAAGAATCG) and TAL-His-Fw (CACCACCACCACCACAGC) for TALEN right subunit. An empty vector strain devoid of TALE-N activity was generated by co-transformation of ScaI-linearized pCT5Ng and pCT6Ng plasmids and used as control in subsequent experiments.

### T7 Endonuclease Test and Sequencing of TALE-N Genetic Targets

Following *M. gaditana* co-transformation with pCT57 and pCT58 and selection on zeocin, obtained colonies harboring both TALE-N subunits were screened by T7 endonuclease assay (T7E1, New England Biolabs, United States), to identify the occurrence of genome editing at the target locus. To this aim, a genomic fragment of 420 bp in the MgAOX1 gene (Naga_100568g3), where the FokI activity region is placed asymmetrically from the 5′ and 3′ ends, was PCR amplified from positive transformants using a Phusion Green proofreading polymerase (ThermoScientific, United States). After electrophoresis of amplified DNA on a 1% agarose gel, bands corresponding to the target MgAOX1 amplicon were purified and quantified. For each clone, 1.3 μg of purified PCR products were treated with T7E1 (+) or left untreated (−). A nontransformed wild-type (WT) colony was also included as a negative control. T7E1 reaction was performed following manufacturer’s instruction, with the following minor modifications. One microliter of T7E1 enzyme was used in each reaction and incubated at 37°C for 1 h. Separation of obtained fragments on 2% agarose gels allowed the detection of mismatched DNA and occurrence of mutations at target sites in some of the clones tested. Positive *M. gaditana* colonies were transferred onto a new selective plate in order to separate cells harboring the mutation from those that presented a WT-like sequence.

Two independent clones were selected based on molecular analysis (Figure 2A): #21 showed a deletion of 10 base pairs (bp); #33 harbored a deletion of 13 bp. Both deletions led to a frame shift in the open reading frame, leading to the appearance of a premature stop codon.

### Photophysiology Measurements

Oxygen exchanges of *M. gaditana* cells were measured with a Clark-type electrode (Hansatech Instruments, United Kingdom) at 20°C. Respiration was quantified by measuring the slope of O_2_ consumption kinetics in the dark. The cyanide-sensitive and -insensitive respirations were calculated from the O_2_ consumption rates using the following relationships:

$$c\,y\,a\,n\,i\,d\,e\,s\,e\,n\,s\,i\,t\,i\,v\,e\,r\,e\,s\,p\,i\,r\,a\,t\,i\,o\,n=\frac{r\,e\,p\,i\,r\,a\,t\,i\,o\,n_{c\,o\,n\,t\,r\,o\,l}-r\,e\,s\,p\,i\,r\,a\,t\,i\,o\,n_{A\,A}}{r\,e\,s\,p\,i\,r\,a\,t\,i\,o\,n_{c\,o\,n\,t\,r\,o\,l}-r\,e\,s\,p\,i\,r\,a\,t\,i\,o\,n_{A\,A\,S\,H\,A\,M}}$$

$$c\,y\,a\,n\,i\,d\,e\,i\,n\,s\,e\,n\,s\,i\,t\,i\,v\,e\,r\,e\,s\,p\,i\,r\,a\,t\,i\,o\,n=\frac{r\,e\,s\,p\,i\,r\,a\,t\,i\,o\,n_{A\,A}-r\,e\,s\,p\,i\,r\,a\,t\,i\,o\,n_{A\,A\,S\,H\,A\,M}}{r\,e\,s\,p\,i\,r\,a\,t\,i\,o\,n_{c\,o\,n\,t\,r\,o\,l}-r\,e\,s\,p\,i\,r\,a\,t\,i\,o\,n_{A\,A\,S\,H\,A\,M}}$$

Antimycin A (AA) blocks the cyanide-sensitive respiratory pathway. Conversely, salicylhydroxamic acid (SHAM) inhibits the cyanide-insensitive respiratory pathway, which is catalyzed by AOX. Both reagents were purchased from Merck (Germany). They were added at the concentrations of 50 μM (AA) and 5 mM (SHAM) from stock solutions in ethanol. Cells were incubated with the inhibitors for 10 min prior to measurements of respiration. We checked that these concentrations were saturating.

Photosynthetic parameters were derived from imaging of chlorophyll fluorescence emission with a Speeedzen III system (JBeamBio, France). Measuring beams were provided by a blue LED source (λ = 470 nm ± 12 nm). Actinic light was generated by an array of red LEDs (emission peak 630 nm ± 40 nm). Acquisition was done with an ORCA flash 4.0 LT camera (Hamamatsu, Japan). The photosynthetic electron transfer rate, ETR_*PSII*_, was calculated as the product of the photon flux density (PFD) in μmol photons⋅m^–2^⋅s^–1^ times the photochemical yield in the light (F_*PSII*_): PFD × (Fm’ - F)/Fm’, where F and Fm″ are the steady-state and maximum fluorescence intensities in light-acclimated cells, respectively (Maxwell and Johnson, 2000). The light intensity was increased stepwise from 29 to 657 μmol photons⋅m^–2^⋅s^–1^. The cells were allowed to reach steady-state fluorescence emissions at each light intensity (time exposure ranging from 5 to 10 min depending on the light intensity) before increasing the photon flux. NPQ was calculated as (Fm – Fm′)/Fm′. Prior to experiments, cells were concentrated at 4 × 10^7^ cells ml^–1^ through centrifugation (3,500 × *g* for 5 min).

### Glycerolipid Analyses

Glycerolipids were extracted from freeze-dried *M. gaditana* cells grown in 50 ml of indicated medium. About 50 to 100 × 10^6^ cells were required for each triplicate analysis. Cells were harvested by centrifugation at 3,500 × *g* for 10 min and immediately frozen in liquid nitrogen and stored at −80°C until use. A freeze-dried pellet was suspended in 4 ml of boiling ethanol for 5 min to prevent lipid degradation, and lipids were extracted as described earlier (Simionato et al., 2013), by addition of 2 ml of methanol and 8 ml of chloroform at room temperature. The mixture was saturated with argon and stirred for 1 h at room temperature. After filtration through glass wool, cell debris were rinsed with 3 ml of chloroform/methanol 2:1, v/v, and 5 ml of NaCl 1% was then added to the filtrate to initiate biphase formation. The organic phase was collected and dried under argon before solubilizing the lipid extract in 1 ml of chloroform. Total glycerolipids were quantified based on their fatty acid (FA) content: in a 50-μl aliquot fraction, a known quantity of 15:0 was added, and FAs were converted into FA methyl esters (FAMEs) by a 1-h incubation in 3 ml of 2.5% H_2_SO_4_ in pure methanol at 100°C (Jouhet et al., 2003). The reaction was stopped by addition of 3 ml of water, and 3 ml of hexane was added for phase separation. After 20 min of incubation, the hexane phase was transferred to a new tube. FAMEs were extracted a second time via addition, incubation, and extraction of another 3 ml of hexane. The combined 6 ml was argon dried and re-suspended in 40 μl of hexane for gas chromatography-flame ionization detector (GC-FID) (Perkin Elmer, USA) analysis on a BPX70 (SGE) column. FAMEs were identified by comparison of their retention times with standards (Sigma, USA) and quantified by the surface peak method using 15:0 for calibration. Glycerolipids were then analyzed and quantified by high-pressure liquid chromatography-tandem mass spectrometry (HPLC-MS/MS), with appropriate standard lipids. The lipid extracts corresponding to 25 nmol of total fatty acids were dissolved in 100 μl of chloroform/methanol [2/1, (v/v)] containing 125 pmol of each internal standard. Internal standards used were phosphatidylethanolamine (PE) 18:0–18:0 and diacylglycerol (DAG) 18:0–22:6 from Avanti Polar Lipid, and sulfoquinovosyldiacylglycerol (SQDG) 16:0–18:0 extracted from spinach thylakoid (Demé et al., 2014) and hydrogenated (Buseman et al., 2006). Lipids were then separated by HPLC and quantified by MS/MS. The HPLC separation method was adapted from previously described procedure (Rainteau et al., 2012). Lipid classes were separated using an Agilent 1200 HPLC system using a 150 mm × 3 mm (length × internal diameter) 5 μM diol column (Macherey-Nagel, Germany), at 40°C. The mobile phases consisted of hexane/isopropanol/water/1 M ammonium acetate, pH 5.3 [625/350/24/1, (v/v/v/v)] (A) and isopropanol/water/1 M ammonium acetate, pH 5.3 [850/149/1, (v/v/v)] (B). The injection volume was 20 μl. After 5 min, the percentage of B was increased linearly from 0 to 100% in 30 min and kept at 100% for 15 min. This elution sequence was followed by a return to 100% A in 5 min and an equilibration for 20 min with 100% A before the next injection, leading to a total runtime of 70 min. The flow rate of the mobile phase was 200 μl min^–1^. The distinct glycerophospholipid classes were eluted successively as a function of the polar head group. Mass spectrometric analysis was performed on a 6460 triple quadrupole mass spectrometer (Agilent, USA) equipped with a jet stream electrospray ion source under the following settings: drying gas heater at 260°C, drying gas flow at 13 L min^–1^, sheath gas heater at 300°C, sheath gas flow at 11 L⋅min^–1^, nebulizer pressure at 25 psi, capillary voltage at ± 5,000 V and nozzle voltage at ± 1,000 V. Nitrogen was used as collision gas. The quadrupoles Q1 and Q3 were operated at the widest and unit resolution, respectively. Phosphatidylcholine (PC) and diacylglyceryl hydroxymethyltrimethyl-β-alanine (DGTS) analyses were carried out in positive ion mode by scanning for precursors of m/z 184 and 236, respectively, at a collision energy (CE) of 34 and 52 eV. SQDG analysis was carried out in negative ion mode by scanning for precursors of m/z −225 at a CE of −56 eV. PE, phosphatidylinositol (PI), phosphatidylglycerol (PG), monogalactosyldiacylglycerol (MGDG), and digalactosyldiacylglycerol (DGDG) measurements were performed in positive ion mode by scanning for neutral losses of 141, 277, 189, 179, and 341 Da at CEs of 20, 12, 16, 8, and 8 eV, respectively. DAG and triacylglycerol (TAG) species were identified and quantified by multiple reaction monitoring (MRM) as singly charged ions [M + NH_4_]^+^ at a CE of 16 and 22 eV, respectively. Quantification was done for each lipid species by multiple reaction monitoring (MRM) with 50-ms dwell time with the various transitions previously recorded (Abida et al., 2015; Dolch et al., 2017). Mass spectra were processed using the MassHunter Workstation software (Agilent, USA) for identification and quantification of lipids. Lipid amounts (pmol) were corrected for response differences between internal standards and endogenous lipids and by comparison with a quality control (QC). QC extract corresponds to a known *Microchloropsis* lipid extract qualified and quantified by TLC and GC-FID as described in Dolch et al. (2017).

## Results

### Mixotrophy in *Microchloropsis gaditana*

Several organic carbon substrates have been reported, so far, to promote mixotrophic growth in *Nannochloropsis* and *Microchloropsis* species, with different efficiencies (Hu and Gao, 2003; Xu et al., 2004a,b; Fang et al., 2004; Sforza et al., 2012; Menegol et al., 2019). As a pre-screen to pinpoint putative mixotrophic substrates in this organism, we searched for possible substrates testing 192 different carbon sources on algal growth simultaneously with Biolog plates (PM01 and PM02A, Bochner, 2003; Stefanowicz, 2006) following an approach already employed in the diatom *P. tricornutum* (Villanova et al., 2017). In this primary screening, most of these compounds inhibited growth in *M. gaditana*, while four (Supplementary Figure 1A, red spots) accelerated growth when compared with a phototrophic control (green line), grown in parallel. These compounds include an amino acid (asparagine) and three organic acids (acetic, fumaric, and malonic), but no sugars.

We sought to identify possible false-positive compounds obtained from this primary screen and tested the ability of the four substrates to promote growth on a larger volume, using 250-ml Erlenmeyer flasks. While asparagine (Supplementary Figure 1B, light blue) had no negative effects when compared with phototrophic conditions (green), the acid compounds significantly inhibited growth. Lowered growth capacity, which has already been reported in *M. gaditana* in the case of acetate (Menegol et al., 2019), was mirrored by a significant inhibition of photosynthetic performances, as evidenced from chlorophyll fluorescence-based measurements of photosynthetic electron flow (ETR_*PSII*_, Supplementary Figure 1C). After several days of growth in the presence of the putative substrates, a significant bleaching of the cells (Supplementary Figure 1D) was observed in the presence of the three organic acids. Overall, these results suggest that this approach to detect mixotrophic substrates is not as powerful in *M. gaditana* as in the diatom *P. tricornutum*, where enhanced growth substrates identified via Biolog plate assays could be confirmed in scaled-up liquid cultures (Villanova et al., 2017).

**FIGURE 1:**
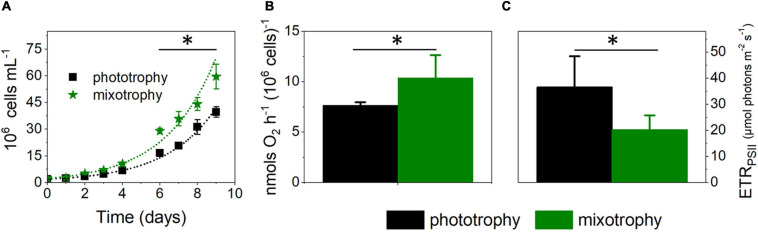
Lysogeny broth boosts growth in *Microchloropsis gaditana*. **(A)** Cell growth is enhanced by addition of 5% w/v Lysogeny broth (LB) to the artificial seawater (ESAW) inorganic medium. Cells were inoculated at a concentration of 2 × 10^6^ cells ml^–1^. From growth traces, we estimated cell division rates of 0.39 ± 0.01 and 0.55 ± 0.06 day^–1^ in phototrophic and mixotrophic conditions, respectively. Number of biological replicates = 4 ± s.d. The asterisk (*) indicates significant differences in cell number (ANOVA, P < 0.05). Dots represent fitting of the data with an exponential growth function. **(B)** LB enhances respiration, measured by dark oxygen consumption. Cells were collected from cultures in exponential growth and concentrated to a density of 4 × 10^7^ cells ml^–1^. Number of biological replicates = 4 ± s.d. The asterisk (*) indicates significant differences in respiration (ANOVA, P < 0.05). **(C)** LB impairs photosynthetic capacity, measured as the electron transport rate (ETR_*PSII*_) parameter at 657 μmol photons⋅m^– 2^⋅s^–1^. Cells were collected from cultures in exponential growth, concentrated to a density of 4 × 10^7^ cells ml^–1^. Number of biological replicates = 4 ± s.d. The asterisk (*) indicates significant differences in photosynthesis (ANOVA, P < 0.05).

In parallel to the Biolog plates trial, we tested other possible mixotrophic substrates. Based on the observation that a peptone-enriched Walne medium is beneficial for cell growth (Marudhupandi et al., 2016) and that addition of Lysogeny broth (LB) improves recovery from transformation of *M. gaditana* cells when added at 5% v/v to the ESAW inorganic medium (G. Allorent, unpublished), we hypothesize that these carbon-rich media may promote mixotrophy. We tested this possibility and found that LB stimulated growth (by 30% ca) when compared with phototrophic conditions (Figure 1A). Addition of LB to *M. gaditana* increased its oxygen consumption capacity (Figure 1B) while leading to a twofold inhibition of photosynthesis, as evinced from the chlorophyll fluorescence-based ETR_*PSII*_ parameter (Figure 1C). Thus, although the reduced carbon added to the cells was beneficial for growth, its presence was detrimental for photosynthetic electron flow capacity.

The LB medium contains amino acids, vitamins, and sugar polymers. Among sugars, a previous work indicated that glucose can implement *Microchloropsis* growth (Xu et al., 2004a,b; Pagnanelli et al., 2014). However, we could not reproduce this result (Supplementary Figure 2) in agreement with recent results (Menegol et al., 2019). We tested the possible role of amino acids and vitamins on *M. gaditana* using another type of Biolog plate (Plate PM05). This plate contains all the amino acids plus four vitamins. We tested growth on the amino acids and vitamins present in this plate and found (Supplementary Figure 3) that none of these compounds could induce a growth benefit to the cells. Thus, we conclude that the effect of the LB on *M. gaditana* cannot be reproduced testing single compounds, suggesting that its benefits on growth may stem from a synergistic effect between the different compounds present in this medium. Alternatively, a compound responsible for the mixotrophic effect of the LB medium could be in insufficient amount in the Biolog plate PM05.

### Generation of Alternative Oxidase Mutants of *Microchloropsis gaditana* by Transcription Activator-Like Effector Nuclease-Mediated Transgenesis

The effect of LB on *M. gaditana* somehow reminds that of exogenous carbon addition to plants, where photosynthesis is often downregulated when respiration is augmented (Koch, 1996; Van Le et al., 2001; McCormick and Kruger, 2015). On the other hand, a positive relationship between respiratory and photosynthetic capacity has been reported in some algae, including *Chlorella* and *Phaeodactylum* (Martínez and Orús, 1991; Bailleul et al., 2015). In diatoms, which belong to Stramenopiles as *Microchloropsis*, knocking down the AOX gene significantly reduced photosynthetic performances and growth (Bailleul et al., 2015), triggering hypersensitivity to oxidative stress (Murik et al., 2019). These findings suggest that the mitochondrial alternative oxidase may play a relevant role in modulating plastid-mitochondrial energy management in this microalgal group. For this reason, we generated AOX mutants in *Microchloropsis* and evaluate their phenotype in both phototrophic and mixotrophic growth conditions.

So far, site-directed mutagenesis was reported for *Nannochloropsis oceanica* via homologous recombination (Kilian et al., 2011; Vieler et al., 2012; Nobusawa et al., 2017, 2019; Gee and Niyogi, 2017) CRISPR/Cas9 (Wang et al., 2016; Poliner et al., 2018; Naduthodi et al., 2019) and RNAi (Wei et al., 2017). In *Microchloropsis* species, site-directed mutagenesis was reported in *M. salina* and *M. gaditana* via homologous recombination (Dolch et al., 2017), CRISPR/Cas9 (Ajjawi et al., 2017; Verruto et al., 2018) and RNAi (Ma et al., 2017; Ajjawi et al., 2017). More recently, mutagenesis based on transcription activator-like effector nucleases (TALE-nuclease) has also been validated in *N. oceanica* (Kurita et al., 2020) and *M. gaditana* (Billey et al., 2021). In this technique, each subunit of the TALE dimer comprises a DNA binding domain and a nuclease domain. The binding domain can be tailored according to a specific recognition code between RVD on the protein and nucleotides of the target sequence. Two subunits of TALE are designed to bind each a target sequence, respectively, on the 5′–3′ strand and 3′–5′ flanking strands so that the nuclease domain induces a double-strand break in the interspace (Sanjana et al., 2012). Nonhomologous end-joining (NHEJ) repair mechanism of the cells is then activated and, in a few cases, leads to misrepair of the break. Mutations issued from failed repair after TALE-N activity can either lead to point mutations in the proteins, to the synthesis of truncated proteins, or to the full loss of protein accumulation (Moscou and Bogdanove, 2009; Miller et al., 2011).

**FIGURE 2:**
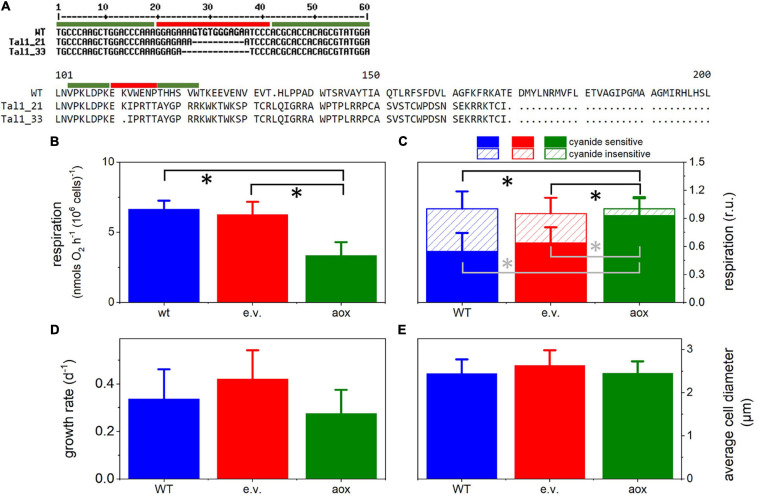
Generation and phenotypic characteristics of *aox* mutants in *Microchloropsis gaditana*. **(A)** Molecular analysis shows the presence of a deletion of 10 bp in clone #21 and of a larger deletion (13 bp) in clone #33, leading to frameshifts in open reading frame and the appearance of premature stop codons. Green bars, specific TALE-N binding sites; red bars, TALE-N Fok1 nuclease cutting site. **(B)** Respiration in the different lines, measured by dark oxygen consumption. Cells were collected from cultures in exponential growth and concentrated to a density of 4 × 10^7^ cells ml^–1^. Asterisks (*) indicate significant differences in respiration (P < 0.05) between the *aox1* and the WT and the empty vector genotypes. **(C)** Cyanide-sensitive and -insensitive respiratory activities in WT and mutant lines. The two pathways were calculated from O_2_ exchange measurements as detailed in the *Methods* section. Asterisks (*) indicate significant differences in the cyanide-insensitive (black) and -sensitive (gray) respiration (P < 0.05) between the *aox1* and the WT and the empty vector genotypes. **(D)** Average cell size estimated from cell imaging with a Luna cell counter. **(E)** Cell division rates, estimated from cell counting with a Luna cell counter. In all panels, the number of biological replicates was 4 ± s.d. WT, wild type; aox, *aox1* knock out mutants (results from clones #33 and #21 were pooled together); e.v., empty vector.

Out of 44 colonies sequenced, 34 harbored deletions at the target site, showing that TALE-nuclease was efficient in more than 75% of the colonies. Two independent clones were selected based on molecular analysis (Figure 2A): #21 showed a deletion of 10 base pairs (bp); #33 harbored a deletion of 13 bp. Both deletions resulted in a frame shift in the open reading frame, leading to the appearance of a premature stop codon.

### Consequences of the *aox1* Mutations in *Microchloropsis* on Algal Physiology

The consequences of knocking-out the AOX1 gene were tested in the two mutants. In these lines, the overall respiratory rate was lower than in the WT, and cells transformed with an empty vector (Figure 2B). To characterize the AOX activity in the WT and mutant strains, we used specific inhibitors of respiration. Antimycin A (AA) blocks the respiratory pathway that does not involve AOX activity, by acting on the cytochrome *bc*_1_ complex. Conversely, salicylhydroxamic acid (SHAM) inhibits the AOX. The AOX activity was quantified as the SHAM-sensitive but AA-insensitive component of respiration (Figure 2C). We found that the AOX contribution, which represented 40–50% of the total dark O_2_ consumption in WT and cells transformed with an empty vector (Figure 2C), was almost completely suppressed in the mutant lines. The lower respiration rates in the mutants suggests that, in these lines, the cyanide-sensitive respiration cannot compensate for the inhibition of the AOX-driven respiration.

**FIGURE 3:**
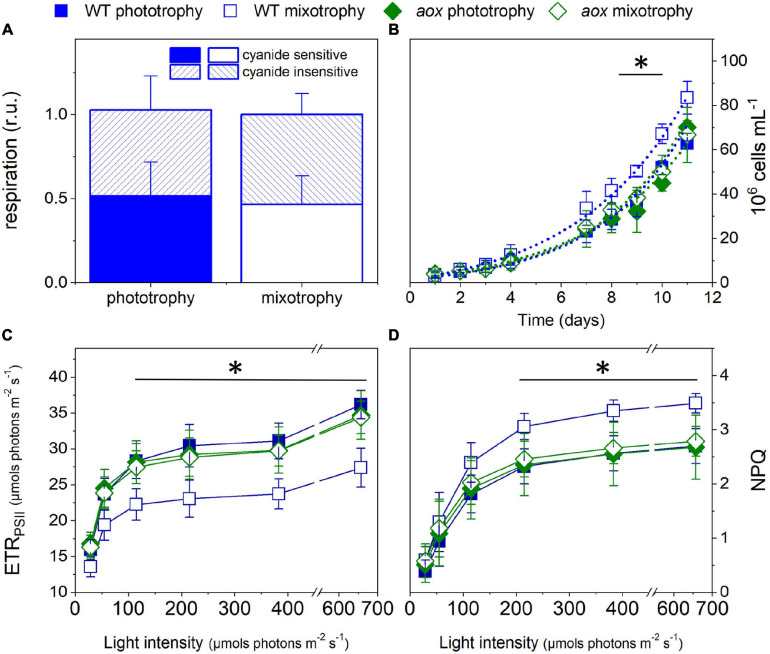
Growth and photosynthetic characteristics of WT and *aox1* mutant strains in phototrophic and mixotrophic conditions. **(A)** Consequences of mixotrophy on AOX activity in WT cells. Cyanide-sensitive and -insensitive respiratory activities were calculated from O_2_ exchange measurements as detailed in the *Methods* section. **(B)** Cell growth measured with a Luna cell counter. From these traces, we estimated division rates of 0.39 ± 0.05 and 0.49 ± 0.04 day^–1^ for WT cells in phototrophic and mixotrophic conditions, respectively, and of 0.40 ± 0.02 and 0.41 ± 0.03 day^–1^ for *aox1* cells in phototrophic and mixotrophic conditions, respectively. The asterisk (*) indicates significant differences in cell number between WT mixotrophy and the other growth conditions (ANOVA, *P* < 0.05). Dots represent fitting of the data with an exponential growth function. **(C)** Light dependence of photosynthetic ETR_*PSII*_. The asterisk (*) indicates significant differences in ETR_*PSII*_ between WT mixotrophy and the other growth conditions (ANOVA, *P* < 0.05). **(D)** Light dependence of non-photochemical quenching (NPQ). The asterisk (*) indicates significant differences in NPQ between WT in mixotrophy and the other growth conditions (ANOVA, *P* < 0.05). In all panels, the number of biological replicates was 4 ± s.d. WT, wild type; aox, *aox1* knock out mutants (results from clones #33 and #21 were pooled together). For experiments in **(C,D)**, cells were collected from cultures in exponential growth and concentrated to a density of 4 × 10^7^ cells ml^–1^.

Unlike diatoms (Bailleul et al., 2015; Murik et al., 2019), AOX mutants in *M. gaditana* did not display any particular cell feature or growth phenotypes, as inferred from their similar growth rates (Figure 3D) and average size of cells in culture (Figure 2E). To further characterize these mutants, we compared their phenotypic traits with those of the WT (Figure 3) in phototrophic (solid symbols) and mixotrophic (empty symbols) conditions. As a prerequisite for these experiments, we tested WT cells for possible changes in the AOX activity between the two conditions, but we could not find any difference between phototrophy and mixotrophy (Figure 3A). We repeated growth experiments with LB (Figure 3B) observing a similar trend as in Figure 1 in the case of WT cells. However, we could not find any effect of LB on growth in the mutants. In the WT, the reduced photosynthetic performances induced by LB (Figure 3C) paralleled with an enhancement of photoprotective responses (NPQ, Figure 3D). We explain this finding by the necessity to compensate for the decreased carbon assimilation through a higher thermal dissipation of excess absorbed photons. Conversely, LB did not diminish the photosynthetic electron transfer in the mutants (Figure 3C) and, therefore, had no effect on its NPQ responses (Figure 3D).

### Effect of Mixotrophy and of Knocking Out the AOX1 Gene on Lipid Metabolism

**FIGURE 4:**
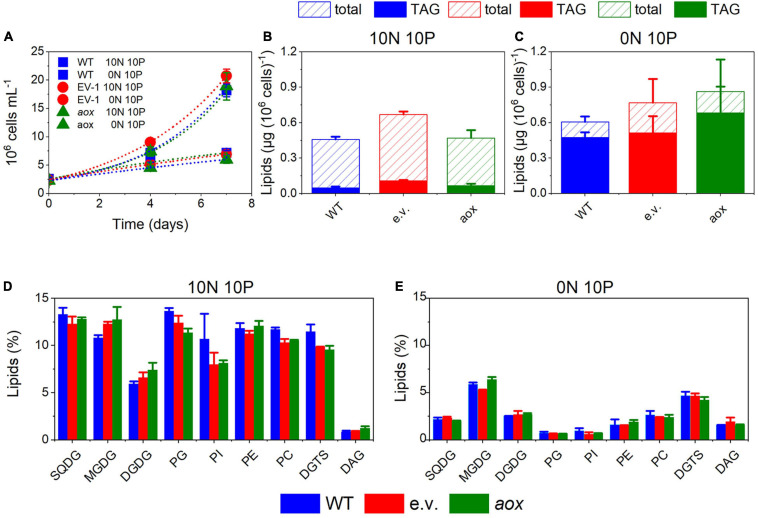
Lipid metabolism in *Microchloropsis* WT and *aox* mutants strains. **(A)** Cell growth under N-replete (10N 10P) and N-deplete (0N 10P) conditions. Dots represent fitting of the data with an exponential growth function. **(B,C)** total lipid and triacylglcerols (TAG) accumulation after 7 days of growth in N-deplete **(B)** and N-replete **(C)** conditions. **(D,E)** Lipid composition in N-replete **(D)** and N-deplete **(E)** conditions. Number of biological replicates = 4 ± s.d. WT, wild type; aox, *aox* knock out mutants (results from clones #33 and #21 were pooled together); e.v., empty vector.

The AOX1 gene is induced in high light-treated *M. gaditana* cells (Alboresi et al., 2016) as well as in nutrient-starved ones (Bailey et al., 2014). Both conditions trigger a remodeling of the lipid composition, enhancing TAG accumulation. Bailey et al. (2014) reported that *Nannochloropsis* mutants with altered AOX levels have a slightly higher content of fatty acid methyl esters (FAMEs). Following this observation, we tested the lipid content and composition of our AOX1 KO lines in both replete and nitrogen-starved conditions. The latter treatment strongly impaired cell growth (Figure 4A). We observed a tendency to increase TAG in the mutants, which, however, turned out not to be statistically significant (Figures 4B,C). The content in polar glycerolipids, mostly represented by monogalactosyldiacylglycerol (MGDG), digalactosyldiacylglycerol (DGDG), sulfoquinovosyldiacylglycerol (SQDG), and phosphatidylcholine (PC) was reduced in nitrogen-depleted cells, although to a different extent depending on the lipid class (Figures 4D,E). We could not observe significant changes in the lipid profiles between the WT and the mutants.

**FIGURE 5:**
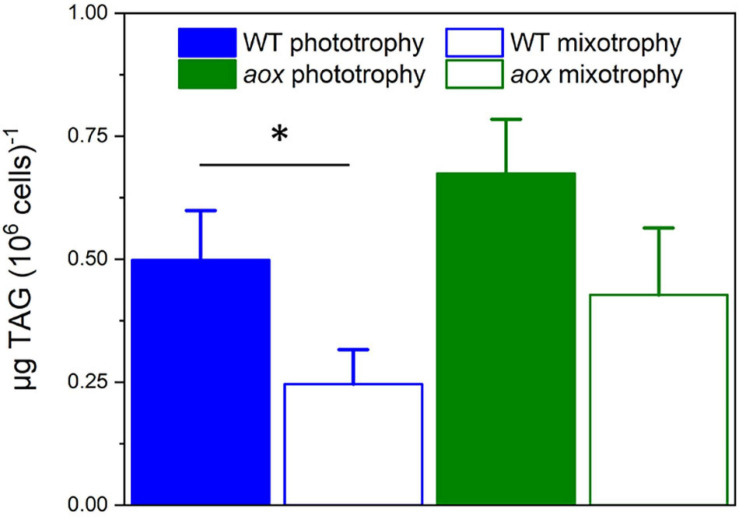
TAG accumulation in *Microchloropsis* WT and *aox* mutants in photoautotrophic and mixotrophic conditions. TAGs were measured 7 days after growth in N-replete (10N_10P) supplemented or not with 5% LB. Number of biological replicates = 4 ± s.d. WT, wild type; aox, *aox1* knock out mutants (results from clones #33 and #21 were pooled together). The asterisk (*) indicates significant differences in TAG content between WT in phototrophic and mixotrophic conditions (ANOVA, *P* < 0.05).

In WT cells, mixotrophic growth triggered a significant reduction of TAG accumulation when compared with phototrophic condition (Figure 5). We interpret this finding in terms of a different cell energy balance between phototrophic and mixotrophic cells. In particular, mixotrophic cells should be more prone to consume energy reserves—likely via fatty acid breakdown through β-oxidation—than phototrophic ones, due to increased respiration and diminished photosynthetic capacity. On the other hand, TAG accumulation was less affected by the shift to mixotrophy in mutant cells, possibly because photosynthetic performances were not diminished by LB addition in the absence of the AOX gene.

## Discussion

In this work, we reinvestigated the consequences of mixotrophy in *M. gaditana*, an alga where this growth mode has been extensively exploited to boost biomass productivity, without a commensurate effort to investigate its physiological and energetic impact on the cell. We first searched for new mixotrophic substrates measuring growth in carbon compound-loaded Biolog plates. We observed enhanced growth on plates with some compounds (asparagine, acetic, fumaric, and malonic acids), but we could not validate the increased growth in liquid cultures, at variance with diatoms (Villanova et al., 2017). Instead, we found that the three acid compounds were detrimental for growth in flasks. This inhibitory effect could reflect an acidification of the cell, thereby preventing carbon assimilation metabolism (e.g., Satoh et al., 2001). Indeed, although the pK of the three acids is rather similar, their inhibition capacity (acetate >malonate >fumarate) is inversely related to the carbon length of their carbon scaffold (two carbon atoms for acetate, there for malonic, and four for fumaric acid). Owing to the relatively low permeability of *M. gaditana* cells to external compounds (see, e.g., Simionato et al., 2013), a smaller carbon scaffold could facilitate the penetration of the cell wall, leading to higher concentrations and, therefore, stronger effects on the cells.

Based on earlier work (Marudhupandi et al., 2016), and our own experiments, biological extracts (peptone or Lysogeny broth) appear as suitable substrates for mixotrophy in *Microchloropsis*. In Marudhupandi et al. (2016), peptone had the largest positive effect on growth among several tested possible substrates. In our case, LB is the only “substrate” that is able to increase growth in liquid cultures to a small extent (compare Figure 1 with Supplementary Figures 1, 2). We employed Biolog plates to pinpoint possible mixotrophic substrates in the LB medium. We found that amino acids could not boost growth when added separately to the phototrophic growth medium (Supplementary Figure 3). This was also true in the case of some vitamins, which could also be present in this medium. These results suggest that the effect of LB on growth may stem from a synergistic effect of the different compounds on *M. gaditana* cells rather than from the effect of a single compound. Alternatively, a compound responsible for the mixotrophic effect of the LB may be in insufficient amount in the Biolog plate PM05.

At the energy metabolism level, the effect of LB is a reduction of the photosynthetic capacity and the enhancement of respiration. This finding indicates that mixotrophic growth in *M. gaditana* is largely dominated by respiratory, rather than photosynthetic energetic metabolism, as already proposed for other algae. In green algae like *C. reinhardtii*, the addition of reduced carbon (acetate) decreases photosynthetic activity and increases respiration, while boosting growth (see Johnson and Alric, 2012; Plancke et al., 2014; Chapman et al., 2015 for further discussion). In other algae, the addition of organic substrates increases respiration without diminishing the photosynthetic capacity. This is, e.g., the case of some *Chlorella* species (e.g., *Chlorella sorokininana*; Cecchin et al., 2018, see, however, Zhang et al., 2017 for a different conclusion in *Chlorella zoofingensis*) and of the diatom *Phaeodactylum tricornutum* (Liu et al., 2009; Villanova et al., 2017). In the green oleaginous alga *Ettlia oleoabundans* and the red extremophyle alga *Galdieria sulphuraria*, mixotrophy stimulates both photosynthesis and respiration (Ferroni et al., 2018; Curien et al., 2021). Overall, these findings indicate that while respiration is always increased by the addition of organic carbon, the photosynthetic responses change according to the nature of the microalga considered. Several interpretations have been proposed for this observation. Increasing respiration could be sufficient to fulfill the cell energy requirement for growth, via, e.g., enhanced consumption of cell reserves, under conditions where contribution by photosynthesis is relatively low. Conversely enhanced respiration could boost photosynthesis by providing extra CO_2_ for carbon assimilation, provided that the photosynthetic apparatus is not dismantled by the addition of exogenous carbon sources (Ferroni et al., 2018; Curien et al., 2021). Finally, respiratory consumption of organic compounds may be incomplete, allowing a (partial) direct utilization of their carbon scaffolds for anabolic processes. This is, e.g., the case of glycerol in *P. tricornutum*, which is respired by the cells, but likely also directly contributes to lipid neosynthesis as a carbon scaffold in mixotrophic cells (Villanova et al., 2017).

An intriguing aspect of the role of respiration in mixotrophy is the role of the mitochondrial alternative oxidase (AOX). While this enzyme generally plays a central role in ROS metabolism in plants, AOX seems to play a more direct role in cell growth in *P. tricornutum* (Bailleul et al., 2015; Murik et al., 2019). Our findings indicate that this is not the case in phototrophic *M. gaditana* cells. However, this enzyme has a role in mixotrophic *M. gaditana* cells because, at variance with the WT (Figure 1), *aox1* mutants do not display any effect on growth, photosynthesis, and photoprotection responses upon addition of LB (Figure 3). This effect somehow reminds previous observations in the *aox-1* mutant of *C. reinhardtii* (Kaye et al., 2019), which displays a severely reduced growth capacity only in mixotrophic conditions. In this alga, two AOX genes exist. While *aox-1* mutants had a strong phenotype, *aox-2* mutants do not show any growth penalty in mixotrophy, suggesting that the two gene products have different functions. In *M. gaditana*, the AOX1 gene duplication often seen in *viridiplantae* (e.g., Neimanis et al., 2013) is not observed. Therefore, the different phenotypes observed in phototrophic and mixotrophic conditions in *M. gaditana* cannot be ascribed to the activity of different AOX enzymes.

A second phenotype related to knocking out the AOX1 gene in *M. gaditana* is at the level of lipid metabolism. In WT cells, changing the balance between respiration (enhanced) and photosynthesis (diminished) upon transitions to mixotrophy leads to a decrease in energy reserve (TAG) accumulation. TAGs are less abundant in mixotrophic cells than in phototrophic ones, suggesting that, this cultivation mode may trigger TAG consumption and FA breakdown (via β-oxidation) to boost cell growth. In the *aox1* mutants, the small growth stimulation by LB is lost and photosynthesis on the, other hand, is not inhibited. It is likely that the different balance between energy production and utilization in the mutant may prevent energy reserves consumption in this genotype. Although at the present state we cannot propose a molecular interpretation for this phenotype, our results clearly indicate that maintaining a proper balance between plastid and mitochondrial activities is a central aspect for energy metabolism and for optimum algal growth in different trophic conditions. This aspect should be carefully considered when trying to optimize the production of microalgal biomass for biotechnology (e.g., lipid accumulation for biofuel) applications (Grama et al., 2016).

## Data Availability Statement

The original contributions presented in the study are included in the article/Supplementary Materials, further inquiries can be directed to the corresponding author/s.

## Author Contributions

DB and MS performed the photophysiology experiments. LM and MB conceived the TALEN. LM constructed the strain, and performed the T7E1 assay, sequencing, and cell banking of the two AOX1 KO strains. LM and EB conducted the molecular characterization of the mutants. ED, CRi, CRa, MoL, and MM developed the growth experiments. MeL, MC, VG, and GT performed the glycerolipid analysis. CG and DF interpreted the data. GC developed the photophysiology experiments. GA interpreted the data and conceived the experiment. DP, FL, and LF conceived the experiment. JJ and EM conceived the experiment and analyzed the data. GF and SC conceived the experiments, analyzed the data and wrote the manuscript. All authors contributed to the article and approved the submitted version.

## Conflict of Interest

EB, FL, LF, and SC are employed by the company Total Refining Chemicals. The remaining authors declare that the research was conducted in the absence of any commercial or financial relationships that could be construed as a potential conflict of interest.

## Footnotes

1 http://www.kazusa.or.jp/codon/
