# Supplementary material for: Consequences of Mixotrophy on Cell Energetic Metabolism in Microchloropsis gaditana Revealed by Genetic Engineering and Metabolic Approaches
Source: Front Plant Sci. 2021 May 25;12:628684. doi: 10.3389/fpls.2021.628684 (PMC8185151; doi:10.3389/fpls.2021.628684)
Supplement: Supplementary file 2 [file Data_Sheet_2.docx]

Supplementary Material

# Supplementary Data

**Supplementary data set 1.** List of the substrates present in the Biolog plates PM01, PM02A and PM05 employed in this work

## Supplementary Figures


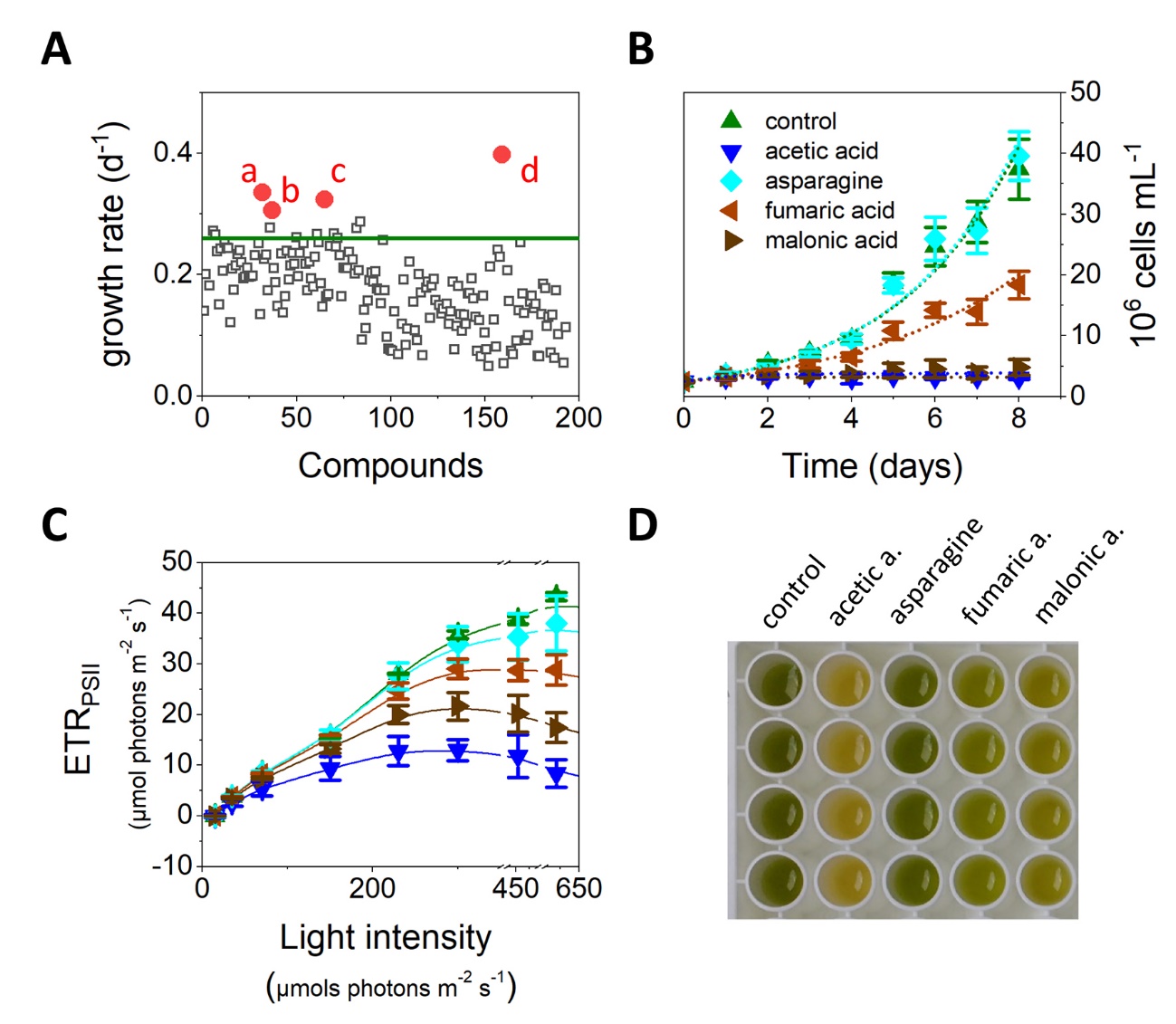


**Supplementary Figure 1.** **Testing mixotrophy in *Microchloropsis gaditana* using Biolog plates**. **A**: cell growth on several organic carbon sources, tested with Biolog plates. Growth was followed for 15 days on 96-wells plates using a Tecan plate reader to estimate division rates from optical density measurements. Compounds (squares) are ordered from 0 (control) to 192 following their order on the Biolog plates (from left to right, top to bottom, see supplementary dataset 1 for a full list of the compounds employed). Data are representatives of an experiment repeated three times with similar results. A few metabolites (red circles) were able to enhance growth when compared to photoautotrophic conditions (green line). These compounds include an aminoacid (asparagine, b) and three organic acids (acetic, a, fumaric, c, and malonic, d). These metabolites were tested in 250 mL Erlenmeyer for growth (**B**) and photosynthetic electron transfer capacity (ETR_PSII_, **C**). **D**: cells collected from Erlenmeyers after 1 week of growth show a significant bleaching in the presence of organic (acetic, fumaric and malonic) acids. Compounds concentration was 5 mM. Acetic, fumaric and malonic acids were added in their acid form. Dots in panel (B) represent fitting of the data with an exponential growth function.


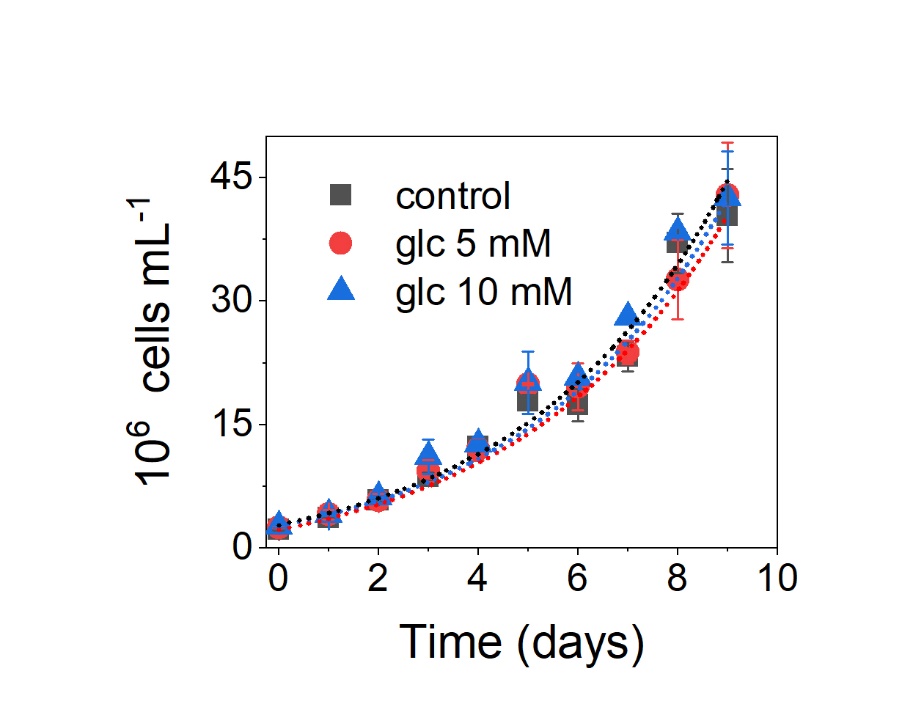


**Supplementary Figure 2. Cell growth is not increased by glucose.** Cell were inoculated at the concentration of 2·10^6^ cells mL^-1^. Experiments were performed as in Figure 2. From these traces, we estimated cell division rates of 0.23 ± 0.09, 0.24 ± 0.11 and 0.25 ± 0.08 days^-1^ for WT cells without glucose, and in the presence of glucose 5 mM and 10 mM, respectively. Dots represent fitting of the data with an exponential growth function.


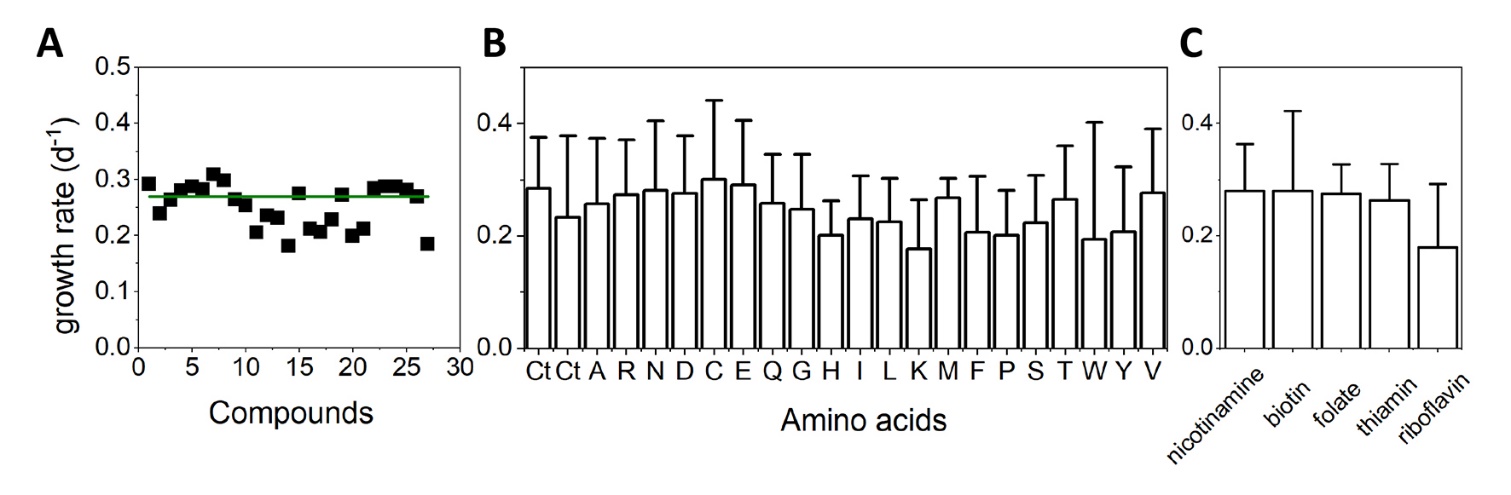


**Supplementary Figure 3 role of aminoacids and vitamins in improving mixotrophic growth in *M. gaditana*.** **A**: cell growth on aminoacids and vitamins, tested with Biolog plates. Growth was followed for 15 days on 96-wells plates using a Tecan plate reader to estimate cell doubling time from optical density measurements. Compounds (squares) are ordered from 0 to 27 according to this order: 1-20: aminoacids; 21-25: vitamins (nicotinamide, biotin, folate, thiamine, riboflavin). Green line: phototrophic conditions. Data are representatives of an experiment repeated six times. **B**: consequences of aminoacids on growth; Same conditions as in panel A. N = 6 from three biological replicates ± s.d. Ct: phototrophic conditions **C:** consequences of vitamins on growth. Same conditions as in panel A. N = 6 from three biological replicates ± s.d.
